# Finding the missed millions: innovations to bring tuberculosis diagnosis closer to key populations

**DOI:** 10.1186/s44263-024-00063-4

**Published:** 2024-05-17

**Authors:** Rachel L. Byrne, Tom Wingfield, Emily R. Adams, Sayera Banu, John Samson Bimba, Andrew Codlin, Ana Cubas Atienzar, Tushar Garg, Stephen John, Ricardo Queiroz Gurgel, Melissa Sander, Victor Santana Santos, S. Bertel Squire, Luan Nguyen Quang Vo, Jacob Creswell

**Affiliations:** 1 Liverpool School of Tropical Medicine, Liverpool, UK; 2 Department of Global Public Health, WHO Collaborating Centre on TB and Social Medicine, Karolinska Institutet, Stockholm, Sweden; 3 Emerging Infections Infectious Diseases Division, International Centre for Diarrhoeal Disease Research, Bangladesh (ICDDR,B), Dhaka, Bangladesh; 4 Zankli Research Centre, Bingham University Karu, Abuja, Nigeria; 5 Friends for International TB Relief, Thanh Xuan, Ha Noi, Vietnam; 6 Stop TB Partnership, Geneva, Switzerland; 7 Janna Health Foundation, Yola, Adamawa State Nigeria; 8 Health Sciences Graduate Program, Federal University of Sergipe, Aracaju, Brazil; 9 Center for Health Promotion and Research, Bamenda, Cameroon

**Keywords:** Tuberculosis, Diagnostics, CAD, TB LAM, Point of care

## Abstract

Current strategies to promptly, effectively, and equitably screen people with tuberculosis (TB) and link them to diagnosis and care are insufficient; new approaches are required to find the millions of people around the world with TB who are missed each year. Interventions also need to be designed considering how people interact with health care facilities and where appropriate should be suitable for use in the community. Here, the historical, new, and reemerging technologies that are being utilised for TB diagnosis globally are discussed, whilst highlighting that how we use and evaluate tests is just as important as the tests themselves.

**Supplementary Information:**

The online version contains supplementary material available at 10.1186/s44263-024-00063-4.

## Background

Tuberculosis (TB) is second only to COVID-19 as the leading cause of single species infectious disease death globally [1], resulting in more than 15 million deaths over the past decade. Due to unprecedented global efforts, multiple public health interventions including vaccinations, diagnostic tests, and therapeutics for treatment and prevention have contained the COVID-19 pandemic. However, the same cannot be said for TB, with deaths still above 2019 levels [2].

TB is a preventable and curable disease with high rates of treatment success for those who are promptly diagnosed and treated. Thus, high TB mortality is often a symptom of diagnostic failures, which contributes to a large portion of the estimated 4 million people with TB who are not diagnosed or notified each year [2].

Historically, most TB strategies and services have been designed at the point of care (POC) for people attending health facilities due to symptoms associated with TB [3]. Patient pathway analyses [4], TB prevalence surveys [5], and research studies [6, 7] indicate that people with TB experience substantial diagnostic delays due to a variety of intersecting personal, societal, and access factors. Key amongst these is as follows: people with TB being asymptomatic, pauci-symptomatic, presenting with atypical symptoms or not recognising their own symptoms, and perceived and enacted stigma and discrimination related to being ill with TB and seeking care. This is further confounded by limitations in health care access relating to distance from health care services and inconvenient opening times, lack of sufficiently trained staff, and the economic impact of seeking care and engaging with TB diagnosis and treatment services. All of which can be associated with catastrophic costs and worsening impoverishment [8, 9]. These are exacerbated by the inequitable distribution of TB, which remains a disease of poverty, concentrated in underserved groups, including children, the urban and rural poor, people living with human immunodeficiency virus (PLHIV), nomads, refugees, prisoners, and internally displaced people. Thus, not only do poorer households have a high burden of TB but also they face the greatest barriers to quality health care access, including individual, community, and structural, and, therefore, are most likely to remain without diagnosis, notification, or treatment [10].

To be diagnosed with TB, a person must first be identified through screening by a health care worker (HCW) and then be offered and receive a diagnostic test. Symptom screening, using the World Health Organization (WHO)-recommended four-symptom screening (W4SS), has long been used by HCWs to identify people with presumptive TB. The W4SS was initially developed to screen PLHIV, for current cough, fever, night sweats, or weight loss [11]. However, prevalence survey data [8] and other studies [12, 13] have suggested that a sizeable proportion of adults with bacteriologically confirmed pulmonary TB, with or without HIV coinfection, screen negative by W4SS. Moreover, it is notable that, even when expanded to include a wider range of possible symptoms, symptom screening still has poor sensitivity to detect TB [5, 8].

Chest X-ray (CXR) is a highly sensitive, symptom agnostic screening tool [14] that has been used to identify people with presumptive TB in high-resource settings over the past century [15]. CXR has higher sensitivity and specificity than symptom screening alone [8, 16] and can potentially reduce the number and costs of follow-on diagnostic tests [16–18]. However, the impact of CXR has been inhibited by a lack of access to equipment and/or radiologists to interpret X-ray images, as well as issues with portability and adaptability in community outreach efforts [13, 19–21].

Current strategies to promptly, effectively, and equitably screen people with TB and link them to diagnosis and care are insufficient; new approaches are required to find the millions of people with TB, around the world, who are missed each year.

## Diagnosing the unreached millions

### Historical approaches

Only a decade ago, almost all microbiological diagnoses of TB were by sputum smear microscopy. Whilst smear microscopy possesses many of the qualities of a desirable diagnostic test — simple, rapid, inexpensive, and specific for highly infectious disease — it has low sensitivity (22-42%) [22], is human resource intensive, incapable of identifying drug resistance, and reliant on operator expertise [23]. Rapid molecular diagnostic testing platforms, such as Cepheid’s GeneXpert^®^ or Molbio’s Trueprep/Truelab^®^ systems, have greatly improved sensitivity [24], but these testing platforms are often unavailable at the point of need (PON). This is largely — despite recent price reductions [25] — due to their high costs, and their requirement for laboratory infrastructure and/or stable electricity, which often confines their deployment to higher-level health care facilities such as district hospitals and referral centres further reducing time to diagnosis. It is therefore not surprising that only 47% of people with TB globally had a rapid molecular assay as their initial TB diagnostic test in 2023 [26].

### New developments and new hope

The COVID-19 pandemic brought global attention to the need for rapid diagnostics and large economic investments in new technologies, as point-of-care (POC) testing substantially reduced turnaround time to strengthen public health responses and achieve epidemic control [27, 28]. We now have a rare opportunity to build on the development and infrastructure of the testing platforms implemented during the COVID-19 pandemic through expansion to address TB. Indeed, many new screening and diagnostic tests are currently being evaluated, and many more envisioned [29]. Further, due to supply chain restrictions, competition for testing capacity, and other resource limitations, pooling of diagnostic specimens was utilised during the COVID-19 pandemic to complement new and repurposed diagnostic platforms, which significantly increased testing capabilities [30–33] and is now being considered for sputum-based TB testing [34].

New products bring choice and a hope of lower costs and better outcomes for people with TB [35]. However, despite the promise of several new assays, we should reflect on lessons learnt from the previous introductions of new tools and temper expectations [36]. Whilst the performance of new tests may be better, and in certain cases cheaper, simply replacing one test with a better performing one does not necessarily lead to more people being diagnosed and treated [24, 37, 38]. Within the complexities of real-world health systems, previous interventions have shown that testing and diagnosing more people with TB require more people to be screened and tested for TB at the PON [39]. We must be cognisant that where we place new testing capacity and how easy it is to access may be equally or potentially more important as the performance profile of the new tests.

## Integrating complementary approaches for TB diagnosis

### Closing the gap: bringing interventions closer to the PON

After years of increasing numbers of people with TB detected through directly observed therapy (DOTS) expansion efforts, TB notifications stalled globally in the middle of the 2000s. A growing realisation that facility-based approaches espoused under the DOTS strategy alone would be insufficient to reach all people affected by TB led to growing interest in outreach strategies. Through the Stop TB Partnership’s TB REACH initiative and other studies, there is extensive evidence suggesting that proactively screening people for TB outside of health facilities, termed active case finding (ACF), can reach more people with TB and earlier in their disease course [40–44]. Based on this evidence, WHO published guidelines on systematic TB screening in 2013 [45] with most high TB burden countries now implementing national strategic plans and receiving Global Fund support for programmatic ACF service delivery. In 2021, the WHO updated and expanded its guidelines for at-risk populations and to include new tools for TB screening [42, 46]. Despite this, the design, implementation, and evaluation of ACF strategies remain highly heterogeneous due to contextual factors specific to individual country settings. Amidst the variability in ACF implementation, the diagnostic tools employed to detect persons with TB serve as a stabilising counterpoint. Specifically, studies have found that when TB diagnostic services are provided closer to where people live and work or at more convenient times, more people with TB can be detected and linked to treatment and care sooner [47, 48]. This early detection should have subsequent positive impact on improving treatment outcomes amongst people with TB, increasing prevention of TB amongst their households, and reducing onward transmission in the community. Community-based ACF approaches have also exhibited socio-protective properties in their capacity to reduce catastrophic costs for people with TB and their households [49, 50]. Such outreach efforts play a critical role in reducing barriers to care seeking and enhancing TB detection amongst key populations [46]. Thus, it is vital that these activities are centred around bringing tests to where people are, rather than being reliant on — and waiting for — people with TB to seek care and attend diagnostic facilities [51].

### Breaking out of the laboratory to expand access to TB diagnosis

Primary health care (PHC) is a major point of entry into health care systems globally. However, inaccurate screening and diagnosis and the subsequent failure to offer relief from illnesses can lead to a breakdown of trust in, and underutilisation of, health services for all diseases, not only TB [52]. Despite an increased focus on the PHC as a cornerstone for meeting the health-related Sustainable Development Goals [53], including achieving Universal Health Coverage, current TB diagnostics are often incompatible with use at PHCs, as they are typically reliant on electricity and laboratory infrastructure. New tools are emerging that are designed for use at the PHC level and in the community. For screening, these repurposed or new tools include ultra-portable digital radiography systems, which can be partnered with computer-aided detection (CAD) software to interpret chest X-ray (CXR) images in the absence of an on-site radiologist, and POC assays including capillary blood C-reactive protein (CRP) tests. For diagnosis, battery-powered molecular testing platforms, lipoarabinomannan (LAM) urine lateral flow assays, and alternatives to sputum specimens, such as upper respiratory swabs and stool specimens, are being considered, with further detail presented below. Timely validation, transparent reporting, and broad dissemination of evaluation results of new diagnostics, including their use in key populations, will be essential to facilitate their adaptation and translation into policy and programmatic implementation.

### POC screening tests

#### Ultra-portable digital radiography systems

**Fig. 1:**
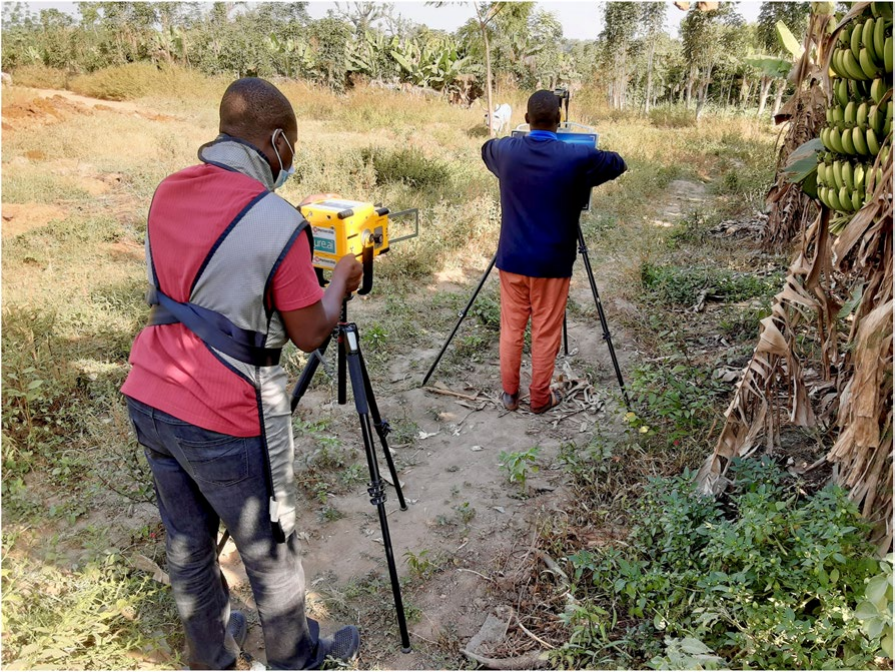
A demonstration of the capabilities of ultra-portable X-ray machines outside healthcare facilities

The development and use of ultra-portable digital radiography equipment represent an opportunity to bring the latest imaging technology closer to the people who need it most [54, 55]. CXR screening for TB requires experienced professionals to read and interpret images. However, in most high TB burden countries, these individuals are limited and typically concentrated in urban centres [20, 21, 55]. The CAD algorithms interpret chest radiographs and provides a TB abnormality score ranging from 0.01 to 0.99 (or 1–100) as an output. This score is derived from the presence and degree of signs consistent with TB (cavitation, consolidation nodules, etc). The higher the score, the greater the radiological involvement and likelihood of the person having TB. The screening programme then can decide at what score a person will be asked to enter into diagnostic testing. Multiple CAD software programmes have been shown to be equivalent or superior to human readers for detecting radiographic abnormalities suggestive of TB in diverse settings making them excellent candidates for deployment in ACF activities [55–57]. CAD software can also be deployed in conjunction with an on-site radiologist as an external quality check on human CXR interpretation (double reading) [58] as a tool to manage workloads, through the triage of normal CXR images [59]; these “use cases” are now becoming common place in mammography screening programmes in high-income countries. Importantly, CAD software is not confined to the standard binary or categorial outcomes used in human-reported CXR readings (e.g. abnormal, suggestive, normal), and continuous variable outcomes can allow programmes to tailor the CAD performance, and thus follow-on testing workloads, based on the needs of each population and the testing capacity at laboratories [60]. This technology is not exclusive to TB and has the potential to be used for many other health-related applications in radiology outside of conventional health care facilities (Fig. 1) [61, 62]. However, high costs of the technology present a barrier to their use at the PON in many high burden countries, yet it is anticipated that as competition and use increase, this will reduce [63]. It is important to note that whilst CAD software programmes have been developed for adults, their utility in paediatric populations has not yet been robustly evaluated, despite one in eight people with TB being a child [64]. As a result, WHO currently only recommends CAD for individuals 15 years old and above [65], which represents a critical knowledge gap for one of the most vulnerable TB target populations.

#### Capillary blood CRP assays

POC assays that detect elevated levels of CRP in capillary blood have been shown to have high sensitivity for TB amongst PLHIV in high burden settings [66]. However, much of the evidence on CRP relates to analytical test performance, and studies evaluating the utility of CRP in key populations and/or clinical settings are scarce [66–68]. To consider their use as a POC screening tool, more evidence is needed in key populations with high TB burden including HIV-negative people. However, it is not only screening tools that should be moving closer to affected people but also diagnostic tests to initiate linkage to care within the PHC and communities.

### POC diagnostic tests

#### Battery-powered molecular diagnostic platforms

Cepheid’s GeneXpert^®^ Omni^™^, despite ultimately being abandoned, exemplified the role battery-powered molecular testing, and mobile solutions could have to reduce laboratory reliance [69] whilst bringing care closer to the PON. There is now a myriad of POC platforms commercially available or underdeveloped that could permit molecular testing to be brought closer to the PON including the Molbio Truenat the first molecular test that can be used at sites with minimal infrastructure to be WHO recommended [70, 71]. Further, because of the increased global investment of polymerase chain reaction (PCR) platforms, there has been an increase of PCR assays for TB able to perform tests for multiple diseases using disposable cartridges at the POC and is now in development for TB [72].

#### Alternatives to sputum specimens

The recommended specimen for most approved TB diagnostic assays is sputum. However, sputum can be difficult to produce and collect from adults and even more so in cases of subclinical TB and from key populations, such as children and PLHIV, whose specimens tend to have lower bacillary loads [73, 74] and who account for a disproportionate share of the missed millions. Further, current alternatives for sputum especially in children tend to be invasive, such as gastric aspirates, and despite stool being a WHO-approved specimen for Xpert testing, uptake remains low [75]. Thus, noninvasive alternatives are needed to ensure all people with TB can be effectively diagnosed. This is represented by an increasing number of publications utilising oral swabs, bioaerosols, and urine for the detection of tuberculosis disease [76–81] as well as AI-based predictive algorithms and cough sound detection technologies. However, all of these diagnostic modalities currently lack sufficient data from prospective clinical studies. A recent systematic review and meta-analysis of the utility of upper respiratory tract samples demonstrated the potential for swabs to expand TB diagnosis. The same report highlighted the lack of prospective studies comparing upper respiratory swabs to sputum testing which are essential to optimise accuracy and sampling strategy in clinical practice [79].

#### Urine LAM lateral flow assays under development

Currently, LAM assay is only recommended for testing in PLHIV with low CD4 counts and report limited sensitivity in the wider population, but newer generation assays are under evaluation, including a third-generation Fujifilm SILVAMP TB LAM II (FujiLAM) which is expected to have much improved sensitivity and could potentially be used amongst people with TB who do not have HIV [78, 82]. Their lateral flow format allows them to be performed at the site of specimen collection without specialised laboratory equipment, which reduces the requirement for transporting specimens and/or patients for results that take time to return and result in loss to follow-up [74].

### Diagnostics in context, not just diagnostic yield

The metrics by which diagnostic tests are evaluated are an important element of understanding their utility in a given population or health system. Test evaluation has historically relied on analytical measures of success, such as sensitivity and specificity, rather than a holistic approach to implementation. In this way, what may be considered an “inferior” diagnostic test in terms of sensitivity and specificity alone may be a hugely useful test if its POC nature allows a greater number of people to be conveniently evaluated. For example, during the COVID-19 pandemic, the analytical sensitivity of lateral flow tests for SARS-CoV-2 did not meet the target product profile (TPP) of a new diagnostic test [83] but still proved to be useful for estimating infectiousness and for taking behavioural steps to avoid onward transmission [84]. With relation to TB, a similar test that can be deployed in PHCs, the community during ACF or even in the home could have great clinical benefit due to its increased proximity to TB-affected people and communities. Additionally, whilst decentralisation of tests offers far-reaching benefits, it is important to address the need for supported quality management systems and oversight that might otherwise be lacking or ill-defined in the PHC.

### Reducing costs to improve access

The global TB community is not on track to reach the goals outlined in the Global Plan to End TB 2023–2030 and the End TB Strategy, in large part due to persistent funding shortfalls [85]. Against this financial backdrop, it is imperative that new initiatives are combined with incentives to reduce overall costs to maximise the impact and global uptake of new diagnostics. One strategy for reducing the cost of testing is through the pooling of diagnostic specimens. Pooling of sputum for TB testing has gained interest following the COVID-19 pandemic where supply chain restrictions, competition for testing capacity, and other resource limitations stimulated interest [30–33]. TB testing yields during ACF are often lower than passive approaches, requiring more testing resources. Pooling specimens is both economically and temporally efficient and enables more people to be tested with molecular diagnostics for the same or less cost; several studies documenting pooling for TB with the Cepheid Xpert assay have shown promising results [86–88].

Another strategy is to reduce the number of referrals for molecular testing by utilising a highly specific, low-cost screening test individually or in combination with others, such as CAD CXR, and/or CRP lateral flow assays. This would facilitate POC screening, and those referred would have a higher likelihood of having active TB disease, who can then be followed up with another diagnostic test that is both sensitive and specific. Such TB diagnostic combination approaches have the potential to reduce the overall costs to both health care systems and individuals, but their effectiveness, cost-effectiveness, feasibility, and acceptability have not yet been evaluated.

The improved performance of TB screening and diagnostic testing alone will not bridge the gap between the 10 million people who develop TB every year and the 7 million who are diagnosed, treated, and notified [38, 89]. We must consider other methods to integrate TB screening and diagnostic testing into other disease service delivery programmes to expand the reach of these new tools and mitigate perceived or enacted stigma of TB health-seeking behaviour.

### Integrating screening and diagnostic services

Programmes and partners delivering TB diagnostic services could work with other disease programmes to mutually extend reach. Vaccine campaigns, nutritional support efforts, maternal child health (MCH) programmes, and others provide opportunities to leverage resources for TB screening efforts with other health initiatives. Whilst TB and HIV services have been integrated for many years [90] and diabetes programmes are beginning to do the same [91], integrating TB services with a wider range of programmes may provide more comprehensive health coverage for the populations they serve at lower marginal costs [92]. Integrating TB with routine lung health screening could produce benefits far beyond infectious diseases. For example, the earlier detection of lung cancer could be a byproduct of routine chest x-rays and increased portability of radiography systems. Further, the use of CAD software as a replacement for local radiologists allows screening to potentially be performed in a decentralized manner [61, 93].

Many patient pathway analyses from low- and middle-income countries (LMICs) have shown the first point of contact with the health system is often private community pharmacies [94, 95]. Non-sputum tests, oral swabs, urine, or others could provide opportunities for testing distribution at these sites [79]. Additionally, one-stop shop ‘test-and-treat’ strategies are also being used by the global HIV community [96] and with developments in testing for and treating TB infection, and disease in a wider approach could provide benefits to the epidemiology at scale if services are combined.

### Community engagement

Beyond the new tools and tests, there is often a critical need to engage key populations to improve the acceptability, feasibility, efficiency, scalability, and sustainability of outreach efforts. Most importantly, this should be done in partnership with the communities where they are intended to be implemented, not only to encourage their participation but to also empower communities to monitor, report, and generate information of their own experiences of TB through community-led monitoring (CLM) [97, 98]. Strengthening community systems therefore is a critical part of efforts to reach all people with TB and supports the development of informed, capable, and coordinated communities and community-based and community-led organisations, groups, and structures [99, 100]. This is now being termed the whole-of-society approach to ensure that the response is equitable, inclusive, people-centred, and promotes gender equality and respects human rights, as outlined during the UN high-level meeting on TB [101].

The whole-of-society approach begins with the investment for TB education, not only to promote awareness of TB symptoms but also to reinforce the message that TB is a preventable and curable disease [2]. The mode of delivery should be tailored to reflect the community in which it is being used and be mindful of local customs, gendered differences, literacy rates, and areas of community congregation. This then continues with the encouragement for people to access health care; this must be made as convenient as possible. Insights can be gained from the HIV community from the success of the ‘know your status’ campaign which improved voluntary testing uptake [102]. Could the TB community embrace a similar approach to change health-seeking behaviour and diagnostic testing provision and uptake? Educational information should then be reinforced at the first encountered health care centre, irrespective of test or treatment availability, about TB, and treatment strategies. Strengthening community engagement with health care centres will not only be helpful to people with TB but will also have wider health benefits [103].

Whilst an engaged and educated community is essential, progress can only go so far without the participation and commitment of policy makers and national governing bodies. The whole-of-government approach encourages TB discussions within all settings, including parliaments, civil society and the educational system, to establish national multisectoral accountability and review mechanisms and increase and sustain investment for community initiatives. Additionally, infrastructure changes which might currently act as a barrier to accessing health care such as opening hours and transportation links from the centres can only be achievable with the combined support of health, finance, trade, and development sectors, in order to enhance collective actions to end TB.

## Conclusions

Despite recent advancements and several promising new technologies in the pipeline, reaching all people with TB with proper diagnosis will not be solved by new tools alone. We must work to bring different combinations of the best tests and use them in creative ways to reach those who are currently being missed by existing TB prevention and care services. This cannot be achieved with a top-down approach, and rather, we must work outside of traditional health facilities to strengthen community systems, collaborating with and looking across disease platforms to reach more people.

Initiatives to target at-risk populations for test evaluation and implementation will provide much needed data on the particular needs of underserved communities whilst contributing to global guidance on finding the missed millions who, each year, are not reached by TB services and care.

## Supplementary Information

Supplementary Material 1.

## Data Availability

Not applicable.

## References

[1] World Health Organization. Global Tuberculosis Report 2021. WHO; 2022. https://www.who.int/publications/i/item/9789240037021. Accessed 29 Aug 2023.

[2] World Health Organization. Global Tuberculosis Report 2022. WHO; 2023. https://www.who.int/teams/global-tuberculosis-programme/tb-reports. Accessed 29 Aug 2023.

[3] Sandiford P. WHO’s DOTS strategy Directly observed therapy. Lancet. 1999;353:755. 10.1016/S0140-6736(05)76125-0. 10073545 10.1016/s0140-6736(05)76125-0

[4] Titahong CN, Ayongwa GN, Waindim Y, Nguafack D, Kuate AK, Wandji IAG, et al. Patient-pathway analysis of tuberculosis services in Cameroon. Trop Med Infect Dis. 2021;6:171. 10.3390/tropicalmed6040171. 34698249 10.3390/tropicalmed6040171 PMC8544654

[5] Aung ST, Nyunt WW, Moe MM, Aung HL, Lwin T. The fourth national tuberculosis prevalence survey in Myanmar. PLOS Global Public Health. 2022;2:e000058810. 10.1371/journal.pgph.0000588. 10.1371/journal.pgph.0000588 PMC10021272 36962394

[6] Odume B, Useni S, Efo E, Dare D, Aniwada E, Nwokoye N, et al. Spatial disparity in availability of tuberculosis diagnostic services based on sector and level of care in Nigeria. J Tuberc Res. 2023;11:12–22. 10.4236/JTR.2023.111002.

[7] Kuupiel D, Cheabu BSN, Yeboah P, Duah J, Addae JK, Ako-Nnubeng IT, et al. Geographic availability of and physical accessibility to tuberculosis diagnostic tests in Ghana: a cross-sectional survey. BMC Health Serv Res. 2023;23:755. 10.1186/S12913-023-09755-3/FIGURES/4. 37452305 10.1186/s12913-023-09755-3 PMC10347710

[8] Onozaki I, Law I, Sismanidis C, Zignol M, Glaziou P, Floyd K. National tuberculosis prevalence surveys in Asia, 1990–2012: an overview of results and lessons learned. Trop Med Int Health. 2015;20:1128–45. 10.1111/TMI.12534. 25943163 10.1111/tmi.12534

[9] Law I, Floyd K, Abukaraig EAB, Addo KK, Adetifa I, Alebachew Z, et al. National tuberculosis prevalence surveys in Africa, 2008–2016: an overview of results and lessons learned. Trop Med Int Health. 2020;25:1308–27. 10.1111/TMI.13485. 32910557 10.1111/tmi.13485 PMC8043149

[10] Oxlade O, Murray M. Tuberculosis and poverty: why are the poor at greater risk in India? PLoS One 2012;7. 10.1371/JOURNAL.PONE.0047533. 10.1371/journal.pone.0047533 PMC3501509 23185241

[11] World Health Organization. Guidelines for intensified tuberculosis case-finding and isoniazid preventative therapy for people living with HIV in resource-constrained settings 2011. WHO; 2012. https://www.who.int/publications/i/item/9789241500708. Accessed 29 Aug 2023.

[12] Ananthakrishnan R, Thiagesan R, Auguesteen S, Karunakaran N, Jayabal L, Jagadeesan M, et al. The impact of chest radiography and Xpert MTB/RIF testing among household contacts in Chennai. India PLoS One. 2020;15:e024120310. 10.1371/JOURNAL.PONE.0241203. 10.1371/journal.pone.0241203 PMC7641361 33147240

[13] Nguyen DTM, Bang ND, Hung NQ, Beasley RP, Hwang LY, Graviss EA. Yield of chest radiograph in tuberculosis screening for HIV-infected persons at a district-level HIV clinic. Int J Tuberc Lung Dis. 2016;20:211–7. 10.5588/IJTLD.15.0705. 26792473 10.5588/ijtld.15.0705

[14] Pinto LM, Pai M, Dheda K, Schwartzman K, Menzies D, Steingart KR. Scoring systems using chest radiographic features for the diagnosis of pulmonary tuberculosis in adults: a systematic review. Eur Respir J. 2013;42:480–94. 10.1183/09031936.00107412. 23222871 10.1183/09031936.00107412

[15] Miller C, Lonnroth K, Sotgiu G, Migliori GB. The long and winding road of chest radiography for tuberculosis detection. Eur Respir J. 2017;49(5):1700364. 10.1183/13993003.00364-2017. 28529204 10.1183/13993003.00364-2017

[16] Nalunjogi J, Mugabe F, Najjingo I, Lusiba P, Olweny F, Mubiru J, et al. Screening for tuberculosis in Uganda: a cross-sectional study. Hindawi. 2021. 10.1155/2021/6622809. 10.1155/2021/6622809 PMC8004368 33828862

[17] van’t Hoog AH, Meme HK, Laserson KF, Agaya JA, Muchiri BG, Githui WA, et al. Screening strategies for tuberculosis prevalence surveys: the value of chest radiography and symptoms. PLoS One 2012;7. 10.1371/JOURNAL.PONE.0038691. 10.1371/journal.pone.0038691 PMC3391193 22792158

[18] Rahman T, Codlin AJ, Rahman M, Nahar A, Reja M, Islam T, et al. An evaluation of automated chest radiography reading software for tuberculosis screening among public-and private-sector patients. Eur Respir J. 2017;49(5):1602159. 10.1183/13993003.02159-2016. 28529202 10.1183/13993003.02159-2016 PMC5460641

[19] Frija G, Blažić I, Frush DP, Hierath M, Kawooya M, Donoso-Bach L, et al. How to improve access to medical imaging in low- and middle-income countries ? EClinicalMedicine. 2021;38:10103410. 10.1016/j.eclinm.2021.101034. 10.1016/j.eclinm.2021.101034 PMC8318869 34337368

[20] Kawooya MG, Kisembo HN, Remedios D, Malumba R, del Rosario Perez M, Ige T, et al. An Africa point of view on quality and safety in imaging. Insights Imaging 2022;13. 10.1186/S13244-022-01203-W. 10.1186/s13244-022-01203-w PMC8959275 35347470

[21] Mbewe C, Chanda-Kapata P, Sunkutu-Sichizya V, Lambwe N, Yakovlyeva N, Chirwa M, et al. An audit of licenced Zambian diagnostic imaging equipment and personnel. PAMJ 2020; 36:32 2020;36:1–14. 10.11604/PAMJ.2020.36.32.21043. 10.11604/pamj.2020.36.32.21043 PMC7388603 32774608

[22] Shin SS, Seung KJ. Tuberculosis. Hunter’s Tropical Medicine and Emerging Infectious Disease: Ninth Edition 2013:416–32. 10.1016/B978-1-4160-4390-4.00039-4.

[23] Desikan P. Sputum smear microscopy in tuberculosis: is it still relevant?. Indian J Med Res. 2013;137(3):442–4. PMC3705651 23640550

[24] Theron G, Zijenah L, Chanda D, Clowes P, Rachow A, Lesosky M, et al. Feasibility, accuracy, and clinical effect of point-of-care Xpert MTB/RIF testing for tuberculosis in primary-care settings in Africa: a multicentre, randomised, controlled trial. Lancet. 2014;383:424–35. 10.1016/S0140-6736(13)62073-5. 24176144 10.1016/S0140-6736(13)62073-5

[25] The Global Fund. Global Fund, USAID and Stop TB Partnership’s new collaboration with Molbio Diagnostics will increase access to rapid molecular tests for TB. The GlobalfundOrg; 2023. https://www.theglobalfund.org/en/news/2023/2023-03-09-global-fund-usaid-stop-tb-partnership-molbio-diagnostics-rapid-molecular-tests-tb/. Accessed 30 Aug 2023.

[26] World Health Organization. Global Tuberculosis Report 2023. WHO; 2023. https://www.who.int/publications/i/item/9789240083851. Accessed 01 Sept 2023.

[27] Brendish NJ, Poole S, Naidu VV, Mansbridge CT, Norton NJ, Wheeler H, et al. Clinical impact of molecular point-of-care testing for suspected COVID-19 in hospital (COV-19POC): a prospective, interventional, non-randomised, controlled study. Lancet Respir Med. 2020;8:1192–200. 10.1016/S2213-2600(20)30454-9. 33038974 10.1016/S2213-2600(20)30454-9 PMC7544498

[28] Iliescu FS, Ionescu AM, Gogianu L, Simion M, Dediu V, Chifiriuc MC, et al. Point-of-care testing—the key in the battle against SARS-CoV-2 pandemic. Micromachines (Basel) 2021;12. 10.3390/MI12121464. 10.3390/mi12121464 PMC8708595 34945314

[29] Pai M, Dewan PK, Swaminathan S. Transforming tuberculosis diagnosis. Nat Microbiol. 2023;8:756–9. 10.1038/s41564-023-01365-3. 37127703 10.1038/s41564-023-01365-3

[30] Regen F, Eren N, Heuser I, Hellmann-Regen J. A simple approach to optimum pool size for pooled SARS-CoV-2 testing. Int J Infect Dis. 2020;100:324–6. 10.1016/j.ijid.2020.08.063. 32866638 10.1016/j.ijid.2020.08.063 PMC7455250

[31] Lohse S, Pfuhl T, Berkó-Göttel B, Rissland J, Geißler T, Gärtner B, et al. Pooling of samples for testing for SARS-CoV-2 in asymptomatic people. Lancet Infect Dis. 2020;20:1231–2. 10.1016/S1473-3099(20)30362-5. 32530425 10.1016/S1473-3099(20)30362-5 PMC7194818

[32] Fogarty A, Joseph A, Shaw D. Pooled saliva samples for COVID-19 surveillance programme. Lancet Respir Med. 2020;8:1078. 10.1016/S2213-2600(20)30444-6. 32976755 10.1016/S2213-2600(20)30444-6 PMC7508511

[33] Liu L. Modeling the optimization of COVID-19 pooled testing: how many samples can be included in a single test? 2022. 10.1016/j.imu.2022.101037. 10.1016/j.imu.2022.101037 PMC9357440 35966127

[34] Vuchas C, Teyim P, Dang BF, Neh A, Keugni L, Che M, et al. Implementation of large-scale pooled testing to increase rapid molecular diagnostic test coverage for tuberculosis: a retrospective evaluation. Sci Rep. 2023;13:15358. 10.1038/s41598-023-41904-w. 37717043 10.1038/s41598-023-41904-w PMC10505184

[35] World Health Organisation. Target product profile for TB screening tests: online public consultation and comment process 2024. https://extranet.who.int/dataformv3/index.php/734221?lang=en (accessed February 23, 2024).

[36] Menzies NA, Cohen T, Lin HH, Murray M, Salomon JA. Population health impact and cost-effectiveness of tuberculosis diagnosis with Xpert MTB/RIF: a dynamic simulation and economic evaluation. PLoS Med. 2012;9:e100134710. 10.1371/JOURNAL.PMED.1001347. 10.1371/journal.pmed.1001347 PMC3502465 23185139

[37] Durovni B, Saraceni V, van den Hof S, Trajman A, Cordeiro-Santos M, Cavalcante S, et al. Impact of replacing smear microscopy with Xpert MTB/RIF for diagnosing tuberculosis in Brazil: a stepped-wedge cluster-randomized trial. PLoS Med 2014;11. 10.1371/JOURNAL.PMED.1001766. 10.1371/journal.pmed.1001766 PMC4260794 25490549

[38] Creswell J, Rai B, Wali R, Sudrungrot S, Adhikari LM, Pant R, et al. Introducing new tuberculosis diagnostics: the impact of Xpert(®) MTB/RIF testing on case notifications in Nepal. Int J Tuberc Lung Dis. 2015;19:545–51. 10.5588/IJTLD.14.0775. 25868022 10.5588/ijtld.14.0775

[39] Joshi B, Lestari T, Graham SM, Baral SC, Verma SC, Ghimire G, et al. The implementation of Xpert MTB/RIF assay for diagnosis of tuberculosis in Nepal: a mixed-methods analysis. PLoS One 2018;13. 10.1371/journal.pone.0201731. 10.1371/journal.pone.0201731 PMC6086427 30096174

[40] Rusen ID, Enarson DA. FIDELIS - innovative approaches to increasing global case detection of tuberculosis. Am J Public Health. 2006;96:14–6. 10.2105/AJPH.2004.056762. 16317206 10.2105/AJPH.2004.056762 PMC1470445

[41] Hinderaker SG, Rusen ID, Chiang CY, Yan L, Heldal E, Enarson DA. The FIDELIS initiative: innovative strategies for increased case finding. Int J Tuberc lung Dis. 2011;15(1):71–6. 21276300

[42] Mhimbira FA, Cuevas LE, Dacombe R, Mkopi A, Sinclair D. Interventions to increase tuberculosis case detection at primary healthcare or community‐level services. Cochrane Database Syst Rev 2017;2017. 10.1002/14651858.CD011432.PUB2. 10.1002/14651858.CD011432.pub2 PMC5721626 29182800

[43] Creswell J, Sahu S, Blok L, Bakker MI, Stevens R, Ditiu L. A multi-site evaluation of innovative approaches to increase tuberculosis case notification: summary results. PLoS One 2014;9. 10.1371/journal.pone.0094465. 10.1371/journal.pone.0094465 PMC3983196 24722399

[44] Burke MRCP RM, A Feasey HR, Ruperez M, Telisinghe MRCP L, Ayles H, Corbett FMedSci EL, et al. Community-based active case-finding interventions for tuberculosis: a systematic review. Articles Lancet Public Health 2021;6:283–99. 10.1016/S2468-2667(21)00033-5. 10.1016/S2468-2667(21)00033-5 PMC8082281 33765456

[45] World Health Organization. Systematic screening for active tuberculosis: principles and recommendations. WHO; 2013. https://iris.who.int/handle/10665/84971. Accessed 05 Sept 2023. 25996015

[46] World Health Organization. Optimizing active case-finding for tuberculosis. WHO; 2021. https://www.who.int/publications/i/item/9789290228486. Accessed 05 Sept 2023.

[47] Shah HD, Nazli Khatib M, Syed ZQ, Gaidhane AM, Yasobant S, Narkhede K, et al. Gaps and interventions across the diagnostic care cascade of TB patients at the level of patient, community and health system: a qualitative review of the literature. Trop Med Infect Dis 2022;7. 10.3390/TROPICALMED7070136. 10.3390/tropicalmed7070136 PMC9315562 35878147

[48] Lorent N, Choun K, Malhotra S, Koeut P, Thai S, Eam Khun K, et al. Challenges from tuberculosis diagnosis to care in community-based active case finding among the urban poor in Cambodia: a mixed-methods study. PLoS One. 2015. 10.1371/journal.pone.0130179. 26222545 10.1371/journal.pone.0130179 PMC4519312

[49] Chandra Gurung S, Rai B, Dixit K, Worrall E, Raj Paudel P, Dhital R, et al. How to reduce household costs for people with tuberculosis: a longitudinal costing survey in Nepal. Health Policy Plan n.d.;2020:1–12. 10.1093/heapol/czaa156. 10.1093/heapol/czaa156 PMC8173598 33341891

[50] Ghazy RM, El Saeh HM, Abdulaziz S, Hammouda EA, Elzorkany AM, Khidr H, et al. A systematic review and meta-analysis of the catastrophic costs incurred by tuberculosis patients. Sci Rep 2022;12. 10.1038/S41598-021-04345-X. 10.1038/s41598-021-04345-x PMC8752613 35017604

[51] Adamou Mana Z, Beaudou CN, Hilaire KFJ, Konso J, Ndahbove C, Waindim Y, et al. Impact of intensified tuberculosis case finding at health facilities on case notifications in Cameroon: a controlled interrupted time series analysis. PLOS Glob Public Health. 2022;2:e000030110. 10.1371/JOURNAL.PGPH.0000301. 10.1371/journal.pgph.0000301 PMC10021155 36962183

[52] Bigio J, MacLean E, Vasquez NA, Huria L, Kohli M, Gore G, et al. Most common reasons for primary care visits in low- and middle-income countries: a systematic review. PLOS Glob Public Health. 2022;2:e000019610. 10.1371/journal.pgph.0000196. 10.1371/journal.pgph.0000196 PMC10022248 36962326

[53] World Health Organization. A vision for primary health care in the 21st century. WHO; 2021. https://www.who.int/docs/default-source/primary-health/vision.pdf. Accessed 15 Sept 2023.

[54] Burke RM, Nliwasa M, Dodd PJ, Feasey HRA, Khundi M, Choko A, et al. Impact of community-wide tuberculosis active case finding and human immunodeficiency virus testing on tuberculosis trends in Malawi. Clin Infect Dis 2023;77. 10.1093/CID/CIAD238. 10.1093/cid/ciad238 PMC10320183 37099318

[55] Pande T, Pai M, Khan FA, Denkinger CM. Use of chest radiography in the 22 highest tuberculosis burden countries. Eur Respir J. 2015;46:1816–9. 10.1183/13993003.01064-2015. 26405288 10.1183/13993003.01064-2015

[56] John S, Abdulkarim S, Usman S, Rahman MdT, Creswell J. Comparing tuberculosis symptom screening to chest X-ray with artificial intelligence in an active case finding campaign in northeast Nigeria. BMC Global and Public Health 2023 1:1 2023;1:1–8. 10.1186/S44263-023-00017-2. 10.1186/s44263-023-00017-2 PMC11622919 39681894

[57] Codlin AJ, Dao TP, Vo LNQ, Forse RJ, Van Truong V, Dang HM, et al. Independent evaluation of 12 artificial intelligence solutions for the detection of tuberculosis. Sci Rep 2021;11. 10.1038/S41598-021-03265-0. 10.1038/s41598-021-03265-0 PMC8668935 34903808

[58] Yoon JH, Han K, Suh HJ, Youk JH, Lee SE, Kim EK. Artificial intelligence-based computer-assisted detection/diagnosis (AI-CAD) for screening mammography: outcomes of AI-CAD in the mammographic interpretation workflow. Eur J Radiol Open 2023;11. 10.1016/j.ejro.2023.100509. 10.1016/j.ejro.2023.100509 PMC10362167 37484980

[59] Lång K, Josefsson V, Larsson AM, Larsson S, Högberg C, Sartor H, et al. Artificial intelligence-supported screen reading versus standard double reading in the mammography screening with artificial intelligence trial (MASAI): a clinical safety analysis of a randomised, controlled, non-inferiority, single-blinded, screening accuracy study. Lancet Oncol. 2023;24:936–44. 10.1016/S1470-2045(23)00298-X. 37541274 10.1016/S1470-2045(23)00298-X

[60] Nguyen L, Vo Q, Banu S, Ahmed S, John S, Health J, et al. Expanding molecular diagnostic coverage for tuberculosis by combining computer-aided chest radiography and sputum specimen pooling: a modeling study from four high burden countries. Research Square (Preprint) 2024. 10.21203/rs.3.rs-3813705/v1. 10.1186/s44263-024-00081-2 PMC11291606 39100507

[61] Liu M, Wu J, Wang N, Zhang X, Bai Y, Guo J, et al. The value of artificial intelligence in the diagnosis of lung cancer: a systematic review and meta-analysis. PLoS One 2023;18. 10.1371/journal.pone.0273445. 10.1371/journal.pone.0273445 PMC10035910 36952523

[62] AI for radiology n.d. https://grand-challenge.org/aiforradiology/ (accessed 26 Oct 2023).

[63] Stop TB partnership. Diagnostics, medical devices & other health products catalog. STP. 2024. https://stoptb.org/assets/documents/gdf/drugsupply/GDFDiagnosticsCatalog.pdf. Accessed 17 Sept 2023.

[64] Geric C, Qin ZZ, Denkinger CM, Kik SV, Marais B, Anjos A, et al. The rise of artificial intelligence reading of chest X-rays for enhanced TB diagnosis and elimination. Int J Tuberc Lung Dis. 2023;27:367–72. 10.5588/ijtld.22.0687. 37143227 10.5588/ijtld.22.0687 PMC10171486

[65] World Health Organization. WHO consolidated guidelines on tuberculosis: Module 5: Management of tuberculosis in children and adolescents. WHO; 2022. https://www.who.int/publications/i/item/9789240046764. Accessed 17 Sept 2023. 35404556

[66] Yoon C, Chaisson LH, Patel SM, Allen IE, Drain PK, Wilson D, et al. Diagnostic accuracy of C-reactive protein for active pulmonary tuberculosis: a systematic review and meta-analysis. Int J Tuberc Lung Dis. 2017;21:1013. 10.5588/IJTLD.17.0078. 28826451 10.5588/ijtld.17.0078 PMC5633000

[67] Lawn SD, Kerkhoff AD, Vogt M, Wood R. Diagnostic and prognostic value of serum C-reactive protein for screening for HIV-associated tuberculosis n.d. 10.5588/ijtld.12.0811. 10.5588/ijtld.12.0811 PMC3816250 23575330

[68] Yoon C, Davis JL, Huang L, Muzoora C, Byakwaga H, Scibetta C, et al. Point-of-care c-reactive protein testing to facilitate implementation of isoniazid preventive therapy for people living with HIV. J Acquir Immune Defic Syndr. 1988;2014(65):551–6. 10.1097/QAI.0000000000000085. 10.1097/QAI.0000000000000085 PMC3999220 24346636

[69] Esmail A, Randall P, Oelofse S, Tomasicchio M, Pooran A, Meldau R, et al. Comparison of two diagnostic intervention packages for community-based active case finding for tuberculosis: an open-label randomized controlled trial. Nature Medicine 2023 29:4 2023;29:1009–16. 10.1038/s41591-023-02247-1. 10.1038/s41591-023-02247-1 36894651

[70] Lee DJ, Kumarasamy N, Resch SC, Sivaramakrishnan GN, Mayer KH, Tripathy S, et al. Rapid, point-of-care diagnosis of tuberculosis with novel Truenat assay: cost-effectiveness analysis for India’s public sector. PLoS One 2019;14. 10.1371/journal.pone.0218890. 10.1371/journal.pone.0218890 PMC6605662 31265470

[71] Stop TB Partnership. Rapid molecular diagnostics for use at peripheral level 2020. https://www.stoptb.org/introducing-new-tools-project/rapid-molecular-diagnostics-use-peripheral-level (accessed 23 Feb 2024).

[72] LumiraDx. LumiraDx announces new investment for ongoing development of its point of care molecular tuberculosis test. https://www.LumiradxCom/Uk-En/News-Events/Lumiradx-Announces-New-Investment-for-Ongoing-Development-of-Its-Point-of-Care-Molecular-Tuberculosis-Test 2022.

[73] Beynon F, Theron G, Respeito D, Mambuque E, Saavedra B, Bulo H, et al. Correlation of Xpert MTB/RIF with measures to assess Mycobacterium tuberculosis bacillary burden in high HIV burden areas of Southern Africa. Sci Rep. 2018;8:1–9. 10.1038/s41598-018-23066-2. 29581435 10.1038/s41598-018-23066-2 PMC5980110

[74] Broger T, Koeppel L, Huerga H, Miller P, Gupta-Wright A, Blanc FX, et al. Diagnostic yield of urine lipoarabinomannan and sputum tuberculosis tests in people living with HIV: a systematic review and meta-analysis of individual participant data. Lancet Glob Health. 2023;11:e903-16. 10.1016/S2214-109X(23)00135-3. 37202025 10.1016/S2214-109X(23)00135-3

[75] World Health Organization. Practical manual of processing stool samples for diagnosis of childhood TB. WHO; 2022. https://www.who.int/publications/i/item/9789240042650. Accessed 08 Oct 2023.

[76] Andama A, Whitman GR, Crowder R, Reza TF, Jaganath D, Mulondo J, et al. Accuracy of tongue swab testing using Xpert MTB-RIF Ultra for tuberculosis diagnosis. J Clin Microbiol 2022;60. 10.1128/JCM.00421-22. 10.1128/jcm.00421-22 PMC9297831 35758702

[77] Patterson B, Dinkele R, Gessner S, Morrow C, Kamariza M, Bertozzi CR, et al. Sensitivity optimisation of tuberculosis bioaerosol sampling. PLoS One 2020;15. 10.1371/journal.pone.0238193. 10.1371/journal.pone.0238193 PMC7470324 32881875

[78] Li Z, Tong X, Liu S, Yue J, Fan H. The value of FujiLAM in the diagnosis of tuberculosis: a systematic review and meta-analysis. Front Public Health. 2021;9:757133. 10.3389/fpubh.2021.757133. 34900905 10.3389/fpubh.2021.757133 PMC8655683

[79] Savage HR, Rickman HM, Burke RM, Odland ML, Savio M, Ringwald B, et al. Accuracy of upper respiratory tract samples to diagnose Mycobacterium tuberculosis: a systematic review and meta-analysis. Lancet Microbe. 2023. 10.1016/S2666-5247(23)00190-8. 37714173 10.1016/S2666-5247(23)00190-8 PMC10547599

[80] Song R, Click ES, McCarthy KD, Heilig CM, McHembere W, Smith JP, et al. Sensitive and feasible specimen collection and testing strategies for diagnosing tuberculosis in young children. JAMA Pediatr. 2021;175:e206069–e206069. 10.1001/JAMAPEDIATRICS.2020.6069. 33616611 10.1001/jamapediatrics.2020.6069 PMC7900937

[81] Laursen LL, Dahl VN, Wejse C. Stool testing for pulmonary TB diagnosis in adults. Int J Tuberc Lung Dis. 2022;26:516–23. 10.5588/IJTLD.21.0305. 35650697 10.5588/ijtld.21.0305

[82] Bjerrum S, Schiller I, Dendukuri N, Kohli M, Nathavitharana RR, Zwerling AA, et al. Lateral flow urine lipoarabinomannan assay for detecting active tuberculosis in people living with HIV. Cochrane Database Syst Rev. 2019;10:CD011420. 10.1002/14651858.CD011420.PUB3. 31633805 10.1002/14651858.CD011420.pub3 PMC6802713

[83] US Food and Drug Administration. Emergency use authorizations for medical devices. 2023 n.d. https://www.fda.gov/medical-devices/emergency-situations-medical-devices/emergency-use-authorizations-medical-devices#covid19ivd (accessed October 20, 2023).

[84] Budd J, Miller BS, Weckman NE, Cherkaoui D, Huang D, Thomas Decruz A, et al. Lateral flow test engineering and lessons learned from COVID-19. Nature Reviews Bioengineering. 2023;1:13–31. 10.1038/s44222-022-00007-3.

[85] Stop TB Partnership. The global plan to end TB. STP; 2023. https://www.stoptb.org/global-plan-to-end-tb/global-plan-to-end-tb-2023-2030. Accessed 08 Oct 2023.

[86] Cuevas LE, Santos VS, Lima SVMA, Kontogianni K, Bimba JS, Iem V, et al. Systematic review of pooling sputum as an efficient method for Xpert MTB/RIF tuberculosis testing during the COVID-19 pandemic. Emerg Infect Dis. 2021;27:719. 10.3201/EID2703.204090. 33622482 10.3201/eid2703.204090 PMC7920689

[87] Santos VS, Allgayer MF, Kontogianni K, Rocha JE, Pimentel BJ, Telma M, et al. Pooling of sputum samples to increase tuberculosis diagnostic capacity in Brazil during the COVID-19 pandemic. Int J Infect Dis. 2023;129:10–4. 10.1016/j.ijid.2023.01.009. 36642209 10.1016/j.ijid.2023.01.009 PMC9834119

[88] John S, Abdulkarim S, Usman S, Rahman MdT, Creswell J. Comparing tuberculosis symptom screening to chest X-ray with artificial intelligence in an active case finding campaign in northeast Nigeria. BMC Glob Public Health. 2023;1:17. 10.1186/s44263-023-00017-2. 10.1186/s44263-023-00017-2 PMC11622919 39681894

[89] Schumacher SG, Thangakunam B, Denkinger CM, Oliver AA, Shakti KB, Qin ZZ, et al. Impact of point-of-care implementation of XpertW MTB/RIF: product vs. process innovation. Int J Tuberc Lung Dis. 2015;19:1084–90. 10.5588/ijtld.15.0120. 26260830 10.5588/ijtld.15.0120

[90] Uyei J, Coetzee D, Macinko J, Guttmacher S. Integrated delivery of HIV and tuberculosis services in sub-Saharan Africa: a systematic review. Lancet Infect Dis. 2011;11:855–67. 10.1016/S1473-3099(11)70145-1. 22035614 10.1016/S1473-3099(11)70145-1

[91] Nyirenda JLZ, Bockey A, Wagner D, Lange B. Effect of tuberculosis (TB) and diabetes mellitus (DM) integrated healthcare on bidirectional screening and treatment outcomes among TB patients and people living with DM in developing countries: a systematic review. Pathog Glob Health. 2023;117(1):36–51. 10.1080/20477724.2022.2046967. 35296216 10.1080/20477724.2022.2046967 PMC9848381

[92] De Foo C, Shrestha P, Wang L, Du Q, García Basteiro AL, Abdullah AS, et al. Integrating tuberculosis and noncommunicable diseases care in low- and middle-income countries (LMICs): a systematic review. PLoS Med. 2022;19(1):e1003899. 10.1371/journal.pmed.1003899. 35041654 10.1371/journal.pmed.1003899 PMC8806070

[93] Koegelenberg CFN, Dorfman S, Schewitz I, Richards GA, Maasdorp S, Smith C, et al. Recommendations for lung cancer screening in Southern Africa. J Thorac Dis. 2019;11:3696–703. 10.21037/jtd.2019.08.66. 31656641 10.21037/jtd.2019.08.66 PMC6790462

[94] Ngure K, Ai-liang Yam E, Nyamuzihwa T, Tembo A, Martyn N, Venter F, et al. The South African community pharmacy sector-an untapped reservoir for delivering HIV services. Front Reprod Health. 2023;5:1173576. 10.3389/frph.2023.1173576. 37519342 10.3389/frph.2023.1173576 PMC10375701

[95] Ilardo ML, Speciale A. The community pharmacist: perceived barriers and patient-centered care communication n.d. 10.3390/ijerph17020536. 10.3390/ijerph17020536 PMC7013626 31952127

[96] Garnett GP, Baggaley RF. Treating our way out of the HIV pandemic: could we, would we, should we? The Lancet. 2009;373:9–11. 10.1016/S0140-6736(08)61698-0. 10.1016/S0140-6736(08)61698-0 19038439

[97] StopTB partnership. ONEIMPACT: for an empowered TB free community. STP; 2023. https://www.stoptb.org/file/17777/download. Accessed 16 Dec 2023.

[98] Stop TB Partnership. Community-led monitoring tools. 2023 n.d. https://www.stoptb.org/digital-health-technology-hub/community-led-monitoring-tools (accessed 23, Feb 2024).

[99] The Global Fund. Technical brief: community systems strengthening. 2019. https://www.theglobalfund.org/media/4790/core_communitysystems_technicalbrief_en.pdf. Accessed 16 Dec 2023.

[100] Kerkhoff AD, West NS, del Mar Castro M, Branigan D, Christopher DJ, Denkinger CM, et al. Placing the values and preferences of people most affected by TB at the center of screening and testing: an approach for reaching the unreached. BMC Global and Public Health 2023 1:1 2023;1:1–16. 10.1186/S44263-023-00027-0. 10.1186/s44263-023-00027-0 PMC11376596 39239641

[101] United Nations. Political declaration of the high-level meeting on the fight against tuberculosis. UN; 2023. https://digitallibrary.un.org/record/4022582?ln=en&v=pdf. Accessed 17 Dec 2023.

[102] Unitaid. Knowing your status- then and now. 2018. https://unitaid.org/assets/STAR-Initiative-Report-Knowing-your-status–then-and-now.pdf. Accessed 18 Dec 2023.

[103] Erku D, Khatri R, Endalamaw A, Wolka E, Nigatu F, Zewdie A, et al. Community engagement initiatives in primary health care to achieve universal health coverage: a realist synthesis of scoping review. PLoS One. 2023;18(5):e0285222. 10.1371/journal.pone.0285222. 37134102 10.1371/journal.pone.0285222 PMC10156058

